# Exploring the barriers and facilitators towards physical activity among church members in Lagos, Nigeria: a qualitative study

**DOI:** 10.4314/ahs.v23i2.66

**Published:** 2023-06

**Authors:** Oluwakemi Ololade Odukoya, Omoladun Olukemi Odediran, Charles R Rogers, Folasade Ogunsola, Kolawole S Okuyemi

**Affiliations:** 1 Department of Community Health and Primary Care, College of Medicine, University of Lagos& Lagos University Teaching Hospital, Idi-Araba, Lagos State, Nigeria; 2 Institute for Health and Equity, Medical College of Wisconsin, Milwaukee, Wisconsin, USA; 3 Department of Microbiology and Parasitology, College of Medicine, University of Lagos, Nigeria

**Keywords:** Physical activity, faith-based organization, determinants

## Abstract

**Background:**

Physical inactivity is substantially linked to the rise in the global burden of non-communicable diseases. Faith-based organizations are recognized as potential partners for sustainable health interventions.

**Objective:**

This study aims to explore the facilitators and barriers towards physical activity among adult church members in Lagos, Nigeria.

**Methods:**

Sixteen focus group discussions (n-163) were conducted among adult male and female church members in twelve Anglican churches. The discussions were audio-taped, transcribed verbatim and analysed along with the field notes for themes using sequential approach with the aid of the Dedoose® software.

**Results:**

Individual facilitators of PA included self-discipline, and personal habits. Individual barriers were laziness, ill-health, fear of injury and pre-existing health conditions. Organizational facilitators included biblical verses promoting PA, while deterring factors were lack of safe spaces for PA and poor knowledge among church leadership. The community-level facilitators included pro-physical activity cultural practices, while the prevailing practice hiring house-helps, high costs of gym membership and gender norms discouraging men from participating in household chores served as deterring community-level factors. Environmental facilitators were the availability of safe spaces for PA while deterring factors were city living and high traffic density.

**Conclusion:**

Several multi-level factors influence physical activity among church members. While it is pertinent to address personal factors, family and community factors also promote PA, therefore, group-level interventions may be warranted. Strategies that address the socio-cultural norms that serve as barriers to PA should also be included in the design of church-based PA programmes.

## Introduction

Globally, 25% of adults and over 80% of adolescents are not sufficiently physically active.^1^ Physical inactivity is one of the leading risk factors for non-communicable diseases (NCDs) such as cardiovascular diseases, several cancers, diabetes, and depression. Regular physical activity improves muscle and blood circulation, as well as bone function and overall health. Moderately intense physical activities such as walking, cycling, and participation in sporting activities have significant health benefits.^2^

Despite the benefits of physical activity, about 21.5% of adults in sub-Saharan Africa are physically inactive.^3^ This contributes to the rise in NCDs in such countries still grappling with the challenges of infectious diseases. As the proportion of urban residents in Africa is predicted to rise to 64% by 2050,^4^ an increase in economic development, coupled with the influence of changing transportation patterns, the use of technology, and eroding cultural values, may contribute to rising levels of physical inactivity.^5^ Besides, several environmental factors linked to urbanization, such as violence, crime in outdoor areas, high traffic congestion, low air quality, lack of parks, and inadequate sports and recreation facilities, can discourage participation in physical activity.^2^ This demonstrates the need for specific interventions to prevent and control physical inactivity as a risk factor for NCDs. For such interventions to be successful, it is imperative to understand the local drivers of these risks in the population. A systematic review exploring the barriers and facilitators for physical activity among adults and older adults of Hispanic and Africa-American minorities noted emergent themes such as the knowledge of the links between physical activity and health, interaction with health professionals, cultural expectations and social responsibilities and conducive environment for physical activity. 6 The study concluded that a huge gap exists in research among Black African groups.

Faith-based organizations provide a unique platform for implementing evidence-based interventions and serve as essential partners in promoting health.^7^ Over the years, faith-based organizations have become a huge part of the African culture. They provide a positive influence and social support for their followers, making these settings valuable venues for sustainable and participatory public health interventions. These institutions can also influence health behaviours at multiple levels (personal levels, family levels, and societal levels), and can reach underserved communities.^8^

Studies in high-income countries among minority ethnic groups such as African-Americans and Hispanics,^9^–^11^ allude to the positive influence of the social and physical church environment and conclude that these may be important factors influencing the physical activity among its members. Several interventions have focused on individual behaviours rather than the church environment and policies or practices, limiting program reach and sustainability.10Limited research exists on interventions to address physical inactivity in faith-based settings in Africa.^12^

As with many African countries, in Nigeria, religion plays an important role and Christianity is one of the major religions in Nigeria.^13^ Conducting a study of this nature may provide data to support a cost-effective and sustainable approach to reducing NCDs' rising burden in Nigeria and several African countries of similar religious beliefs. In addition, the information obtained from our study may be helpful to other religious African countries design faith-based interventions for physical activity.

**Figure 1:**
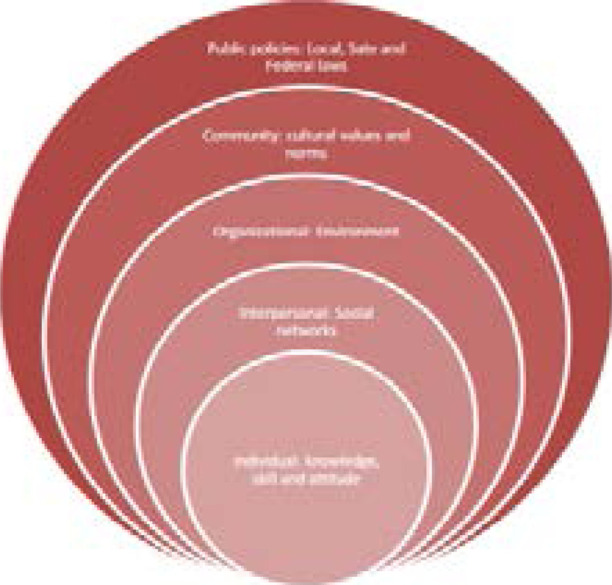
The Social Ecological Model

This paper forms part of a larger study that explores the opinions of church members and church leaders on the determinants of physical activity. We used the Social Ecological Model (Figure 1), which considers the individual, their interrelationships with others, their community and the spiritual and organizational contexts to explore the factors associated with participation in physical activity.^14^,^15^ The knowledge obtained would provide valuable insights into developing tailored and culturally appropriate interventions for sustainable behavioural change in faith-based settings.

## Methods

### Study design and participants

The study design and methods of the main study have been previously described.^12^ In summary, participants were members of a large orthodox group of churches located in Lagos, Nigeria. We collected data from November 2018 to December 2018 using focus group discussions. Eligible participants for the focus groups were men and women between the ages of 18 and 80 years, residents in Lagos, Nigeria, and church members who had attended the church for at least one year and attended church services weekly. The participants were selected purposively.

The study team, which included church leaders and members, developed the initial guides informed by the Social Ecological Model (SEM)for the focus group and assured that draft questions were in line with study objectives. These questions were then pretested among a separate group of church members of the same denomination. The questions were subsequently modified to ensure they were biblically, culturally and linguistically appropriate before use.

### Recruitment, data collection, and setting

The study team worked with the church leaders to identify and recruit eligible participants by word of mouth. Focus group sessions were held within the church premises and facilitated by trained moderators and note-takers who were all holders of Master of Science in Public Health led by O.O.*They were also trained on preventing assumptions and personal biases from influencing participants' responses. Each team had a facilitator, two note-takers, and three teams conducted the FGDS.O.O.^*^ is the lead researcher, a female, Medical Doctor and an Associate Professor at the College of Medicine, University of Lagos. We held focus group sessions in three distinct participant age groups a) 18-30 years b) 31-65 years c) >65 years of age. Sessions began with a welcome/introductory message, informing participants of the purpose and nature of the study, obtaining informed consent and assuring them of confidentiality. We collected basic demographic information such as age and sex and assigned codes to each participant. Each session lasted between 45 and 90 minutes and we collected data until a saturation point was reached. Saturation point was defined using base size, taken as the point at which no new information was obtained from the FGDs.16 In total 16 FGDs were held. Only research team members and recruited participants were present at the discussions. Discussions were audio-taped and field notes taken. A total of 163 people participated in the FGDs.

Discussions were conducted only once and all the discussions were conducted in the English language and duly recorded.

### Data Analysis

After removing personal identifiers, all recordings were transcribed verbatim by at least two independent trained research assistants. Led by the lead researcher [OO], we adopted a sequential approach to analyse the data.16This was done by initially applying a deductive approach where we drew initial codes from the existing literature on the topic of inquiry aided by the research aims and objectives, coded the data into categories. Subsequently, we used an inductive approach to derive additional codes from the data and merged both sets of codes. Then, we uploaded the codes and transcripts onto DedooseTM, assigned relevant codes to the quotes within each transcript, and summarized the data for presentation using the Social Ecological Model (SEM). Finally, the notes' content was integrated with the transcription from the discussion before analysis.

### Ethical Consideration

Ethical approval was obtained from the Research Ethics Committee of the College of Medicine, University of Lagos, with the approval number CMUL/HREC/05/18/347.

## Results

### Sample characteristics

Sixteen focus group discussions each averaging 75 minutes were conducted from November 2018 to December 2018; eight sessions were among adults aged 31-64, four sessions among ages 18-30 and another four sessions among church members that are 65 year and older. Study's participants' mean age was 47.6years. The table 1 provides additional characteristics of the participants in the focus group discussion.

**Table 1:** Socio-demographic characteristics of the respondents, (n-163) in the focus group discussions at Anglican Churches Lagos mainland, Nigeria. Held from November 2018 to December 2018

Focus Group Sessions	Number of sessions	Number of participants	Mean age (SD)	Male: Female distribution
Youth (18-30 years)	4	46	23.1(3.5)	25:21(54.3%:45.7%)
Adults (31-64 years)	8	72	46.8(10.2)	24:28(33.3%:66.7%)
Elderly (65 years or older)	4	45	74.3(5.2)	15:30(33.3%:66.7%)
Overall	16	163	47.7(20.6)	69:94(42.3%:57.7%)

The barriers to and facilitators for physical activity are summarized in the table 2

**Table 2:** Facilitators and barriers to physical activity as mentioned by the respondents of a focus group discussion carried out at Anglican Churches, Lagos Mainland, Nigeria. Held from November 2018 to December 2018

Themes	Facilitators	Barriers
Individual determinants	*Self-discipline, personal habits, self-awareness and improved appearance*	*Laziness and lack of interest, ill-health, fear of injury and discomfort often felt at the start of a new exercise regimen, poor mental health, preexisting health condition and delayed gratification*

Interpersonal determinants	*Support received from other family members, presence of young children in the home, the desire not to become a liability to one's children* and *family habits*.	*Grief due to the loss of a family member*

Organizational determinants	*Biblical verses promoting spiritual physical activities like dancing and discourage laziness*.	*Lack of space provided by the church for physical activity, poor knowledge of the importance of physical activity among church leaders* and *focus on secondary health prevention often tailored to the elderly*

Community determinants	*Certain cultural practices*	*The prevailing practices of employing hired helps in the home, high costs of gym memberships*, and *gender norm which* discourages men from participating in household chores

Public policy determinants	Availability of safe spaces	*Inadequate space, city living, and high traffic density, and insecurity*.

### Individual determinants of physical activity

The most prevalent theme was that physical activity is beneficial for everyone.

*‘Yes, when you wake up in the morning, it is good for the body to run around. It is good for the body.’ (FGD Adult female)*

Self discipline and habit cultivated are perceived to be important facilitators for physical activity. Some youth also see physical activity as an opportunity to improve their aesthetic appearances. People also participate in physical activity because they desire to look good as well as the knowledge of the benefits of physical activity.

*“…the idea of not wanting to be fat makes me wants to be physically active” (FGD Youth male)*

Recurrent sub-themes for personal deterring factors were laziness or lack of interest, fear of injury, the discomfort often felt at the start of an exercise regimen, poor mental and physical health, and delayed gratification. However, the fear of getting injured while participating in physical activity was mentioned especially among the elderly.

*“Laziness makes people to be less physically active.” (FGD adult male)*

*“The first time I started, I was too eager to go round the whole process, you start with jogging and switch over to press-ups straight up, but…the next day I felt like a log of wood. No, oh! The pain was excruciating…seriously! It discouraged me for a while…” (FGD youth male)*

*“I am afraid of exercise due to fear of injury. One has to be careful not to fall at this age”. (FGD Elderly male)*

Poor mental health such as depression and the general lack of interest associated with it and some pre-existing health conditions such as bone or joint problems, asthma, and epilepsy were also mentioned by respondents.

*“Happiness encourages physical activity but if you are depressed, you won't be physically active.” (FGD adult female)*

*“…health condition…for example if you have arthritis, you cannot walk or if the person is asthmatic, he cannot jump because he is prone to seizures and therefore, you cannot do things that can affect yourself. So, it depends on your health condition.” (FGD adult male)*

When the motivation for physical activity is primarily weight loss, the long lag between engagement in physical activity and the achievement of weight loss goals may also serve as a determinant to consistent engagement in physical activity. One respondent noted that the lack of a perceived change in her weight by her observers demotivated her from continuing with her physical activity plans.

### Interpersonal determinants of physical activity

Motivational support from family members may serve as a facilitator of physical activity. Household heads may set good examples for other family members and serve as a source of motivation for engaging in physical activities. A respondent stated:

*“…like in my home, my husband will wake up before 6 a.m. walking around the compound, that encourages me to wake up and walk around the compound.” (FGD elderly female)*

The elderly also noted that the absence of growing children in the home may reduce the motivation and encouragement for physical activity among the elderly.

Family motivations for physical activity among the elderly included the desire to avoid becoming a liability to their children. An elderly respondent opined as follows:

*“I exercise so that I will not become a liability to my children”. (FGD elderly)*

Deterring factors mentioned by participants were family issues which may also serve as a deterrent to engagement in physical activity, for instance the chronic illness or the loss of a family member.

*“Someone that lost her husband or if the children her sick, the person won't be happy and won't be physically active.” (FGD Adult male)*

### Organizational determinants of physical activity

Respondents noted that several bible characters exhibited great physical strength and some stories in the bible depict physical activity such as “marching around the walls of Jericho.” Also, certain church routines such as kneeling, singing and dancing to God were noted to encourage physical activity.

*“We have people in the bible known for their physical strength like Samson”. (FGD adult female)*

However, some respondents lamented the inability of the church to provide facilities for physical activity for church members. One respondent said:

*“Church should provide physical amenities and should add information on healthy eating and physical activity in the bulletin.” (FGD youth male)*

The youths stated that most health-promoting activities in the church were directed at the elderly and are primarily towards the secondary prevention of diseases.

A youth respondent said about the health programs: *“We don't have youth represented at meetings. Many of our health programs focus on the elderly.” (FGD youth male)*

### Community determinants of physical activity

Some respondents alluded to the fact that festivals and cultural activities such as farming and dancing promote physical activity. *“…for instance, we come from this part of Africa and major occupation for us is farming.” (FGD elderly male) “Well, our cultural music will make you dance” (FGD youth male)*

Deterring factors mentioned by the participants include; the common practice of hiring helps in the home which may promote sedentary behaviour, especially among the elderly. A respondent said, “and when you reach certain age the children feel that you must have somebody…” (FGD elderly female)

Also mentioned is the high cost of payment for gym memberships being a deterrent to participation in physical activity outside the home. According to a respondent,

*“I cannot afford to go to the gym but rich people can do so. (FGD adult male)*

Gender norms may contribute towards physical activity among women as socio-cultural expectations of women often make them engage in house chores which tend to increase their physical activity levels,

*“If you are a housewife, you will be physically active because you cook, clean and tidy the house”. (FGD elderly female)*

Also, male dominance and the social expectations of men being served by women may encourage women to be more active compared to men as expressed by a participant:

*“If you are a wife, you will be physically active because you have to cook for daddy.” (FGD elderly female)*

There was a general submission that advancing age and male gender were associated with declines in physical activity as male elders are more prone to sedentary lifestyle since they are not involved in performing household chores due to their societal status both as men and as elders in the Nigerian culture.

An elderly male respondent said, *“When I finish eating and pack my plate, my wife will say if the children see me, they will say they are not taking care of me.” (FGD elderly male)*

### Policy determinants of physical activity

Most respondents reported that living in a safe neighbourhood with available infrastructure for physical activity was a promoting factor. Some respondents opined that the lack of safe spaces for exercise and the challenges that accompany urban living may serve as barriers to outdoor physical activity.

*“There is no space for exercise. The roads are full of okadas (commercial motorcyclists). The environment where I live does not encourage one to exercise”. (FGD adult male)*

High traffic density associated with city living was also said to deter the engagement in physical activity.

*“Sometimes traffic because sometimes when you get home you are always tired and you have to sleep” (FGD youth male)*

Some respondents noted that the fear of being attacked by hoodlums while engaging in physical activity outside the home serves as a deterring factor.

*“I cannot wake up early in the morning to do exercise due to the kind of environment I'm living. I could be attacked by armed robbers.” (FGD adult female)*

## Discussion

This study aimed to gain insights into the determinants of physical inactivity among church members in a faith-based setting prior to implementing a church-based program designed to increase physical activity. The complex interplay between individual, environmental, family, sociocultural, and spiritual/religious determinants impacted the participants' decisions to engage in physical activity.

Few studies have been done on the barriers and facilitators to physical activity among church members. Most of these were conducted in high-income countries, did not employ qualitative techniques and did not use a theoretical framework such as the Social Ecological Model.^7^,^9^,^17^Onestudy carried out in various Catholic and Protestant churches with large Latino membership in San Diego County, California, used the consolidated framework for implementation research to inform a semi-structured interview guide,^7^ the study concluded that community cooperation and denominational support are important for an effective intervention for physical activity in faith-based settings. Our study adds to this knowledge by elucidating the specific enabling and deterring factors across levels, to be considered for implementing a successful and sustainable intervention for physical activity in church settings.

We noted that individual-level factors such as self-discipline and inculcating physical activity habits during adolescence often influence decisions to be involved in physical activity. For example, a study across Minnesota, United States, in 2018 revealed that about 22% of adults reported a lack of self-discipline as a deterrent to being more physically active.^18^ In a representative population survey carried out in Taiwan using the exercise habits questionnaire by sports administration of the ministry of education; researchers collected information from 413 people, age from 25 to 65years on their adolescent and adult exercise habits, demographic data, and chronic diseases details. The study noted that adolescent exercise habits correlated positively with adults' exercise habits, adults with exercise habits also have better physical performance, and exercise habits of adults must be had been established since adolescence.^19^ Our findings suggest that the perception of the importance of physical activity in adults may result from habits formed when younger.

Ill health, such as bone problems, the fear of injury, and poor mental health, served as deterrents to physical activity. Physical inactivity is linked to poor bone health and osteoporosis. Therefore, increased physical activity at any stage of life has a positive effect on bone health and should be encouraged among study participants.^20^,^21^

Interpersonal factors, such as adequate support from family and friends, are cited as motivation for physical activity. In contrast, family problems such as health, loss of family, and the absence of growing children were deterrents. Similar studies were observed among college staff in the Mid-Western United States, where family responsibilities, lack of family support, and scheduling were perceived as barriers. At the same time, facilitators noted were available family support, the presence of young people and children active in the home, and the need to work as a role model for these children.^22^ Similar findings were also noted among minority ethnic groups in the United Kingdom, where life factors such as the time spent at work, household demands, and cost implications of enrolling in exercise classes served as significant barriers to physical activity.^23^

Lack of facilities like open spaces and gyms for physical activity in the church premises was viewed as a deterrent to physical activity in our study. Pre-existing church culture and norms, alignment with church mission and values, competing priorities, lack of resources, and the need for buy-in from senior leadership were noted as barriers to physical activity among the church members in another study.^7^ Our study suggests that religion may play a role in adopting positive health behaviours as it was noted that some bible scriptures promote physical activity. This may serve as a possible link between faith-based interventions and positive health outcomes. 24

Gender norms and the differences in social roles attributed to men and women as they advance in age served as key sociocultural determinants of physical activity. In the Nigerian culture, men are perceived as household heads. They are often spared from the daily household chores resulting in an increased likelihood of physical inactivity among men.^25^,^26^ Women, on the other hand, tend to have traditional roles that involve household chores like cooking and cleaning in the home. Although the women in our study viewed daily household chores as a form of physical activity, this is in contrast to the opinions of women in more developed climes where these responsibilities were noted as deterring factors to physical activity among women.^27^,^28^

Regardless of the influence of individuals and social factors on decisions to participate in physical activity, the physical environment is also important in promoting participation in physical activity.^6^,^29^ In particular, the lack of safe spaces, congested roads, and high traffic density in urban areas are clear factors mentioned by the respondents in our study. Young adults in Bangladesh also point to the lack of neighbourhood safety, poor street lighting at night, lack of free spaces to exercise, and traffic congestion coupled with non-motorized public transportation as environmental barriers to physical activity.^29^,^30^ These findings are consistent with a previous study among Nigerians where perceived crime and neighbourhood safety from traffic were identified as important deterrents to physical activity engagement.^31^

Our study was primarily exploratory and conducted prior to a physical activity intervention. Similar pre-intervention studies for physical activity in faith-based settings have been conducted in high-income countries with different socio-economic and religious contexts. Furthermore, we employed the SEM, which explores barriers and facilitators not limited to the individual levels but also the family and societal levels. Research across these levels provides a detailed understanding of these contexts and how best to address/optimize them for a successful and sustainable intervention. Our FGDs were also conducted across three distinct age groups, enabling a more thorough understanding of how these factors may or may not differ within the different population groups. Finally, some of the barriers mentioned by participants in our study are culturally peculiar, providing insights to inform the design of such interventions in similar sociocultural settings. A similar study was conducted in four urban and rural churches in South Africa^17^ to evaluate the feasibility, acceptability and potential effectiveness of healthy living and not physical activity in particular, and it was not an exploratory study.

The insights obtained from this study provide information about contextual factors to inform the design of interventions targeted at church members. We identified specific barriers such as delayed gratification, laziness and health issues. Others are socio-cultural barriers such as high costs of a gym membership, gender norms, and the prevailing practice of hiring house-helps. Environmental barriers include high traffic density, inadequate safe spaces, and insecurity and some organisational barriers, such as the inability of the church to provide facilities for physical activity and the focus of the church on secondary prevention of diseases. We identified facilitators such as self-discipline, habit formation from youth, the presence of young children in the household, role modelling, the desire not to become a liability to one's children in old age. Biblical verses which support physical activity and discourage laziness should also be explored to encourage sustained participation in physical activity.

These insights will inform the design of interventions. For example, the health talks and educational materials will be designed to dispel the common myths regarding physical activity and incorporate specific ways to address cultural and physical barriers. Family and community level interventions which strengthen facilitators and address barriers at the at the family/community levels will also be explored. Leveraging on scriptural support for physical activity to encourage biblical values that promote self-discipline and build motivation as well as spiritual<=““ span=”” style=“font-family: “Times New Roman”;”>-physical activities such as dancing will also be employed. Educating the church leadership on the importance of physical activity as primary prevention for NCDs and the need to prioritize the provision of safe spaces for PA for the church members are recommended.

Despite this study's contributions, the findings should be interpreted with some caution. First, the purposive nature of participant sampling and the fact that respondents were drawn from a single church denomination makes generalisation to all church members or to churches of alternative denominations or other religions impossible. However, the relatively large sample (n=163) and the well-spread age distribution of the selected participants provide a robust knowledge base and key insights into the determinants of physical activity prior to the implementation of such programs in religious settings.

In conclusion, our results revealed several multi-level factors which may influence physical activity among church members. This insight may be useful in designing interventions for PA in similar settings. Personal factors, family and community influence PA, therefore, individual and group-level interventions may be warranted. Addressing the socio-cultural norms that serve as barriers to PA should be included in the design of church-based PA programs. Incorporating scriptural values and biblical verses that promote PA into the design of such programs is also recommended.
